# Looking like *Limulus*? – Retinula axons and visual neuropils of the median and lateral eyes of scorpions

**DOI:** 10.1186/1742-9994-10-40

**Published:** 2013-07-11

**Authors:** Tobias Lehmann, Roland R Melzer

**Affiliations:** 1 SNSB – Bavarian State Collection of Zoology, Münchhausenstraße 21, Munich 81247, Germany; 2 Department Biology II, Ludwig-Maximilians-Universität München, Großhaderner Straße 2, Planegg-Martinsried 82152, Germany; 3 GeoBio-Center at LMU, Richard-Wagner-Str. 10, 80333, Munich, Germany

**Keywords:** Chelicerata, Scorpiones, Visual system, Central projections, Phylogeny

## Abstract

**Background:**

Despite ongoing interest in the neurophysiology of visual systems in scorpions, aspects of their neuroanatomy have received little attention. Lately sets of neuroanatomical characters have contributed important arguments to the discussion of arthropod ground patterns and phylogeny. In various attempts to reconstruct phylogeny (from morphological, morphological + molecular, or molecular data) scorpions were placed either as basalmost Arachnida, or within Arachnida with changing sister-group relationships, or grouped with the extinct Eurypterida and Xiphosura inside the Merostomata. Thus, the position of scorpions is a key to understanding chelicerate evolution. To shed more light on this, the present study for the first time combines various techniques (Cobalt fills, DiI / DiO labelling, osmium-ethyl gallate procedure, and AMIRA 3D-reconstruction) to explore central projections and visual neuropils of median and lateral eyes in *Euscorpius italicus* (Herbst, 1800) and *E. hadzii* Di Caporiacco, 1950.

**Results:**

Scorpion median eye retinula cells are linked to a first and a second visual neuropil, while some fibres additionally connect the median eyes with the arcuate body. The lateral eye retinula cells are linked to a first and a second visual neuropil as well, with the second neuropil being partly shared by projections from both eyes.

**Conclusions:**

Comparing these results to previous studies on the visual systems of scorpions and other chelicerates, we found striking similarities to the innervation pattern in *Limulus polyphemus* for both median and lateral eyes. This supports from a visual system point of view at least a phylogenetically basal position of Scorpiones in Arachnida, or even a close relationship to Xiphosura. In addition, we propose a ground pattern for the central projections of chelicerate median eyes.

## Introduction

Scorpions have two classes of eyes: one pair of large elevated eyes in the middle of the carapace commonly referred to as median eyes, and two to five pairs of small eyes along the anterior, lateral margin of the carapace, commonly referred to as lateral eyes [1]. In both types, the eye is composed of a cuticular lens, photoreceptor cells, arhabdomeric cells, efferent neurosecretory fibres, and pigment cells. However, there are characteristic differences in ultrastructure: in the lateral eyes the focusing lens and the vitreous body are lacking, and the rhabdomeres of all retinula cells form a contiguous rhabdom; median eyes, on the other hand, possess a focusing lens and a vitreous body, and the rhabdomeres of 4-6 retinula cells form separated star-shaped rhabdoms [2-4]. Additionally a pair of minute accessory lateral eyes have been demonstrated in prenymphs and nymphs of *Parabuthus transvaalicus* at the posterior end of the lateral eye row, and separated from these by a cuticular ridge [5]. These eyes are composed of photoreceptor cells, arhabdomeric cells and efferent neurosecretory fibres, but a cuticular lens and pigment granules are absent.

The median eyes are the scorpion's main eyes, allow good image processing with relatively high acuity and good spatial discrimination, and exhibit a distinct circadian sensitivity rhythm [3]. In lateral eyes, due to the construction of the dioptric apparatus as well as the anatomy of the retina, the visual acuity is reduced. They have been suggested to function mainly as extremely sensitive light detectors, e.g. for *Zeitgeber* stimuli to synchronize a circadian clock [3,4]. The neurobiology of this circadian clock is well known for the North African desert scorpion, *Androctonus australis* (see summary by Fleissner [6]).

So far, the visual systems of scorpions have been studied mainly in a neurophysiological context, whereas their morphological features are undescribed on a level that would allow phylogenetic comparisons [7-9]. Holmgren [7] suggested a series of four visual neuropils (“Seemasse 1–4”), with the median eyes linked to the first and the lateral eyes to the second neuropil. Holmgren’s pupil, Hanström [8], identified the same neuropils, but distinguished between median and lateral eye neuropils and suggested that the median eyes are linked to one neuropil and the lateral eyes to three subsequent neuropils, while some fibre bundles project from the median eye neuropil to the third lateral eye neuropil. Fleissner [9] reported that the photoreceptor cell axons of the median eyes terminate within a first neuropil (“lamina”), while the axons of the arhabdomeric cells terminate in a second neuropil (“medulla”); the retinula cell axon terminals of the lateral eyes were not defined.

Lately the structure and development of various nervous systems have played important roles in debates concerning arthropod evolution and phylogeny. For this field of research two different approaches – “neurophylogeny” [10,11] and “neural cladistics” [12] – were established.

In Chelicerata other than Scorpiones, especially well studied visual systems are that of the xiphosuran *Limulus polyphemus*[13-16], which is an important, well investigated species in the field of visual neuroscience, and those of several Araneae [17-20] (*Salticus scenicus*, *Habrocestum pulex*, and *Cupiennius salei*). Recent investigations addressed visual systems in Pycnogonida [21]*(Achelia langi*, *A. vulgaris*, and *Endeis spinosa*), the sister taxon to Euchelicerata or even to Euarthropoda, and in Onychophora [22,23] (*Euperipatoides rowelli*, *Epiperipatus biolleyi*, and *Metaperipatus blainvillei*), a putative arthropod outgroup.

The phylogenetic position of Scorpiones was discussed in various ways over the last one hundred years: Analysis based on morphological data either saw Scorpiones as highly ancestral Arachnida and as the sister taxon to Lipoctena (= all other arachnids) [24], or grouped Scorpiones together with Opiliones, Pseudoscorpiones, and Solifugae, to form the arachnid subgroup of Dromopoda [25]. Recently five arachnid clades were proposed, one of them being the clade Stomothecata comprising Scorpiones and Opiliones, but the relationships between those 5 clades is unresolved [26]. Combined morphological and molecular analyses support Dromopoda [27,28]. Also in molecular studies, the phylogenetic position of scorpions is interpreted in different ways [29,30]. And lastly some palaeontologists continue a long tradition of placing scorpions outside Arachnida with Eurypterida [31,32]. Eurypterida (the sea scorpions) is the extinct sister taxon to Xiphosura, with which it forms the group Merostomata.

To make the visual system in scorpions accessible for phylogenetic comparison with those in other chelicerates, the present study employs several independent approaches (3D serial reconstruction, Cobalt fills, DiI / DiO labelling, Wigglesworth stains). In the scorpion species *Euscorpius italicus* (Herbst, 1800) and *E. hadzii* Di Caporiacco, 1950, the visual neuropils of the median and lateral eyes are identified with Cobalt fills and DiI / DiO labelling, and their general architecture is studied along with the termination sites of retinula cell axons. Additionally the main neuropils of the protocerebrum are described by means of osmium-ethyl gallate procedure and AMIRA 3D-reconstruction. This reveals features of the visual system generally studied in Chelicerata, to allow comparisons with other lineages.

## Results

### General layout of the visual system

**Figure 1:**
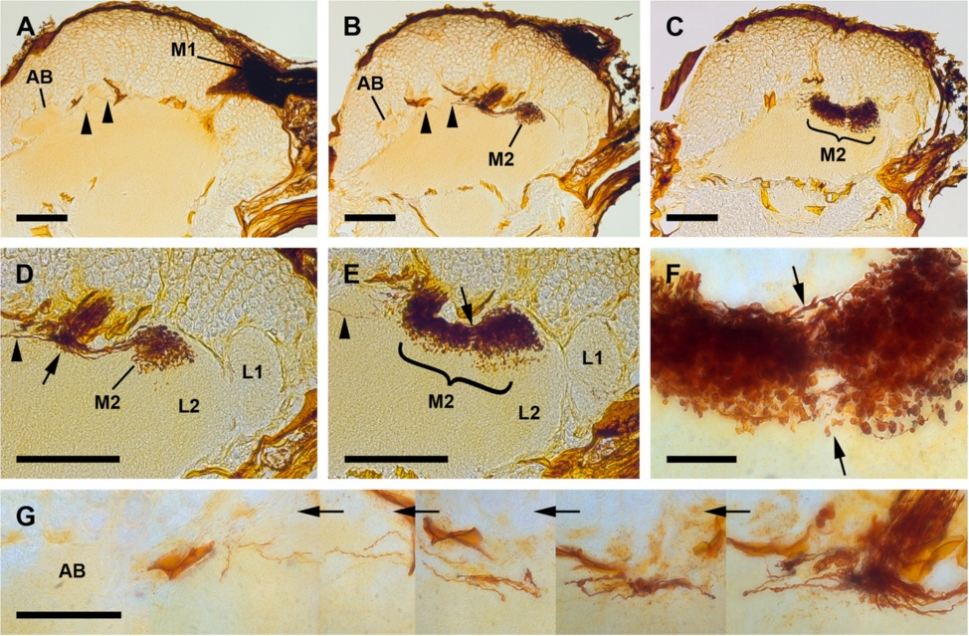
**Cobalt fills via median eyes, sagittal sections. A**, first median eye visual neuropil posteriorly in dorso-lateral protocerebrum. Note dense arrangement of Cobalt-filled profiles. Arrowheads point to axons extending to arcuate body. Bar 100 μm. **B**, bifurcation of fibres projecting from first to second median eye neuropil. Arrowheads point to axons extending to arcuate body. Bar 100 μm. **C**, second median eye visual neuropil under cell body rind, divided by an annulus into posterior and anterior subunit. Bar 100 μm. **D**, detail of bifurcation, varicosities in anterior subunit of second median eye visual neuropil. Arrowhead points to axons extending to arcuate body. Bar 100 μm. **E**, detail of second median eye visual neuropil, showing varicosities in both subunits. Arrowhead points to axons extending to arcuate body. Bar 100 μm. **F**, detail of annulus (arrows). Bar 25 μm. **G**, combination of five successive sections to demonstrate path of Cobalt-filled axons connecting median eyes with arcuate body via bifurcation seen in B and D. Bar 50 μm. AB, arcuate body; L1, first lateral eye visual neuropil; L2, second lateral eye visual neuropil; M1, first median eye visual neuropil; M2, second median eye visual neuropil.

The visual system in the studied scorpion species, *Euscorpius italicus* and *E. hadzii*, is composed of two median eyes located medially on top of the cephalothorax, and two pairs of lateral eyes located along the front corners of the cephalothorax. Nerve fibres project from the median and lateral eyes proximally to the dorso-lateral protocerebrum. The two median eyes supply two distinct, successive visual neuropils as targets of the R-cell axons; few fibres additionally connect the median eyes with the arcuate body (Figure 1). The first neuropil is located dorso-anteriorly in the lateral part of the protocerebrum, as an oval-shaped region laterally embedded in the cell body rind of the brain (Figure 1A). The second neuropil lies deeper, under the cell body rind and in a more ventral and lateral position in the protocerebrum (Figure 1B–F).

**Figure 2:**
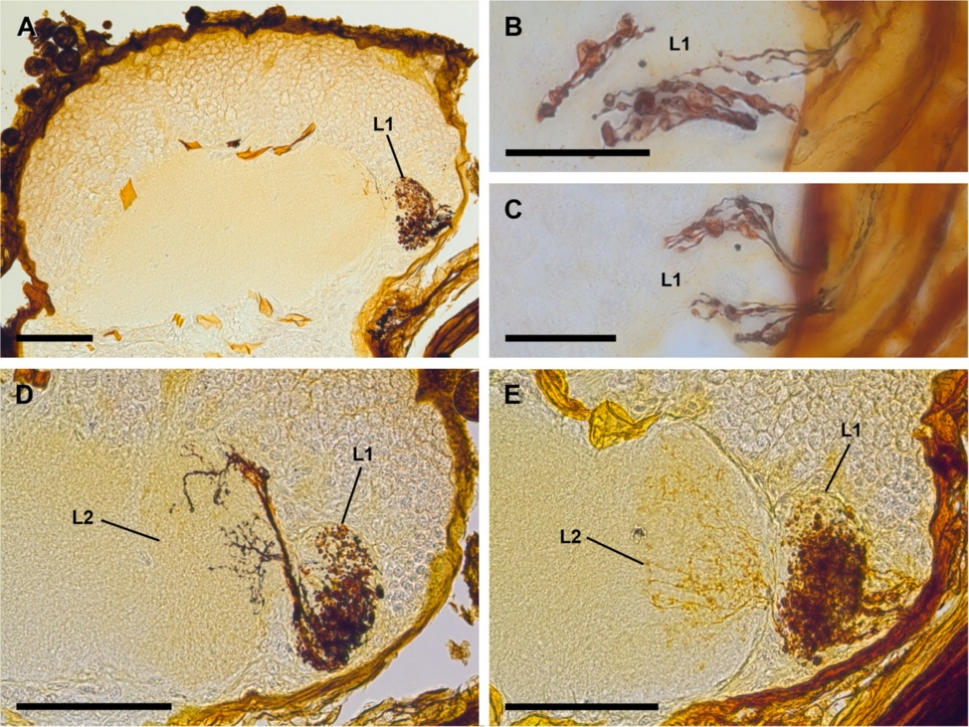
**Cobalt fills via lateral eyes, sagittal sections. A**, first lateral eye visual neuropil posteriorly in ventro-lateral protocerebrum. Note dense arrangement of Cobalt-filled profiles. Bar 100 μm. **B**, **C**, details of Cobalt-filled retinula axons with varicosities at entrances to first lateral eye visual neuropil. Bars 25 μm. **D**, **E**, Cobalt fills of retinula axons terminating in first and second lateral eye visual neuropils. Note that some fibres seem to cross between first and second visual neuropils. Bars 100 μm. L1, first lateral eye visual neuropil; L2, second lateral eye visual neuropil.

**Figure 3:**
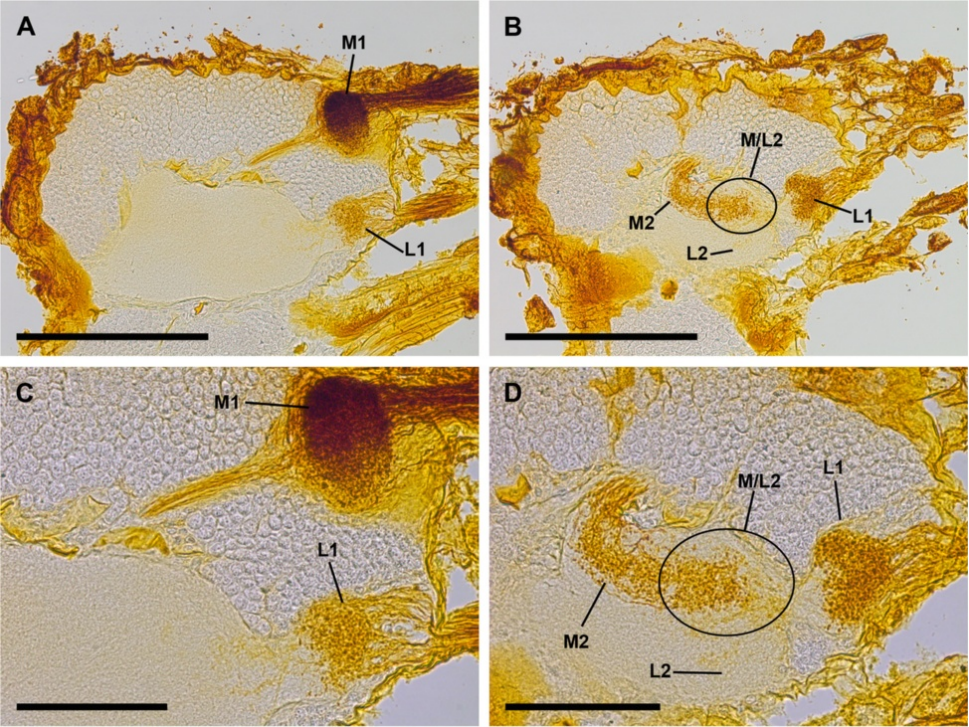
**Cobalt fills simultaneously via median and lateral eyes, sagittal sections. A**, first median and lateral eye visual neuropils, located posteriorly in lateral protocerebrum. Both neuropils with Cobalt-filled retinula axons. Bar 250 μm. **B**, second visual neuropils of median and lateral eyes. Besides regions with only Cobalt-filled R-cell axons of median or lateral eyes, encircled region with Cobalt-filled R-cell axons of both median and lateral eyes. Bar 250 μm. **C**, detail of first median and lateral eye visual neuropils. Note tract through cell body rind projecting to second median eye visual neuropil. First varicosities appear posterior to first lateral eye visual neuropil, indicating second lateral eye visual neuropil. Bar 100 μm. **D**, detail of second visual neuropils of median and lateral eyes. One can distinguish between lateral and median eye fills, lateral fills brighter. Besides regions with only Cobalt-filled R-cell axons of median or lateral eyes, encircled region with Cobalt-filled R-cell axons of both median and lateral eyes. Bar 100 μm. L1, first lateral eye visual neuropil; L2, second lateral eye visual neuropil; M1, first median eye visual neuropil; M2, second median eye visual neuropil; M/L2, region of L2 or M2 with Cobalt-filled R-cell axons of both median and lateral eyes.

**Figure 4:**
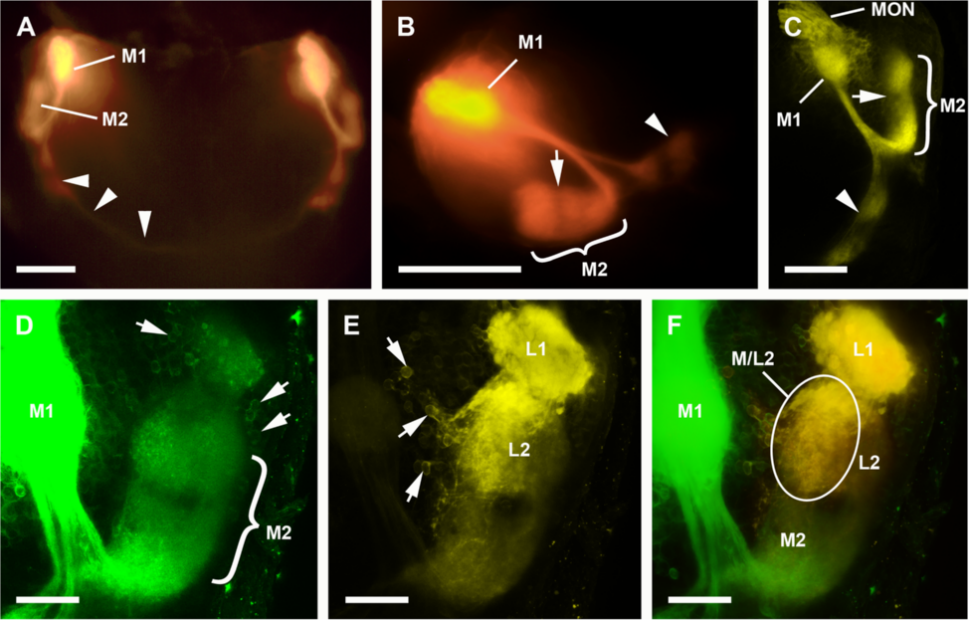
**DiI and DiO labelling via median or median and lateral eyes (A, B, fluorescence microscope; C–D, CLSM). A**, **B**, DiI-labelled first and second median eye neuropils. Arrowheads point to axons extending to arcuate body with varicosities after bifurcation, few axons terminating within the arcuate body. Note same annulus as seen in Cobalt fills (arrow). **A**, frontal view; **B**, sagittal view. Bars 200 μm. **C**, Specimen as in **A**, **B**, studied with CLSM. Frontal view. Bar 100 μm. **D**–**E**, Combined DiO-labelled median (green) and DiI-labelled lateral (yellow) eye neuropils. Frontal view. Bars 50 μm. **D**, DiO-labelled first and second median eye neuropils (green). Note that DiO-stained cell bodies (arrows) indicate transcellular staining. **E**, DiI-labelled first and second lateral eye neuropils (yellow). Note that DiI-stained cell bodies (arrows) indicate transcellular staining. **F**, Combined image of DiO-labelled median (green) and DiI-labelled lateral (yellow) eye neuropils. Encircled region of second median and lateral eye neuropils with labelled R-cell axons of both median and lateral eyes. L1, first lateral eye visual neuropil; L2, second lateral eye visual neuropil; M1, first median eye visual neuropil; M2, second median eye visual neuropil; M/L2, region of L2 or M2 with labelled R-cell axons of both median and lateral eyes; MON, median eye optic nerve.

The two lateral eyes also supply two distinct, successive visual neuropils as targets of the R-cell axons (Figure 2). The second visual neuropils of the median and lateral eyes overlap each other; this means that some R-cell axons of the median and lateral eyes end in a shared region of the second visual neuropil (Figures 3, 4). The first neuropil is located in the lateral and anterior part of the protocerebrum, 50–100 μm ventrally underneath the first visual neuropil of the median eyes. It is oval, and laterally embedded in the cell body rind of the brain (Figure 2A–C). The second neuropil lies posterior to the first neuropil and is also oval (Figure 2D, E).

**Figure 5:**
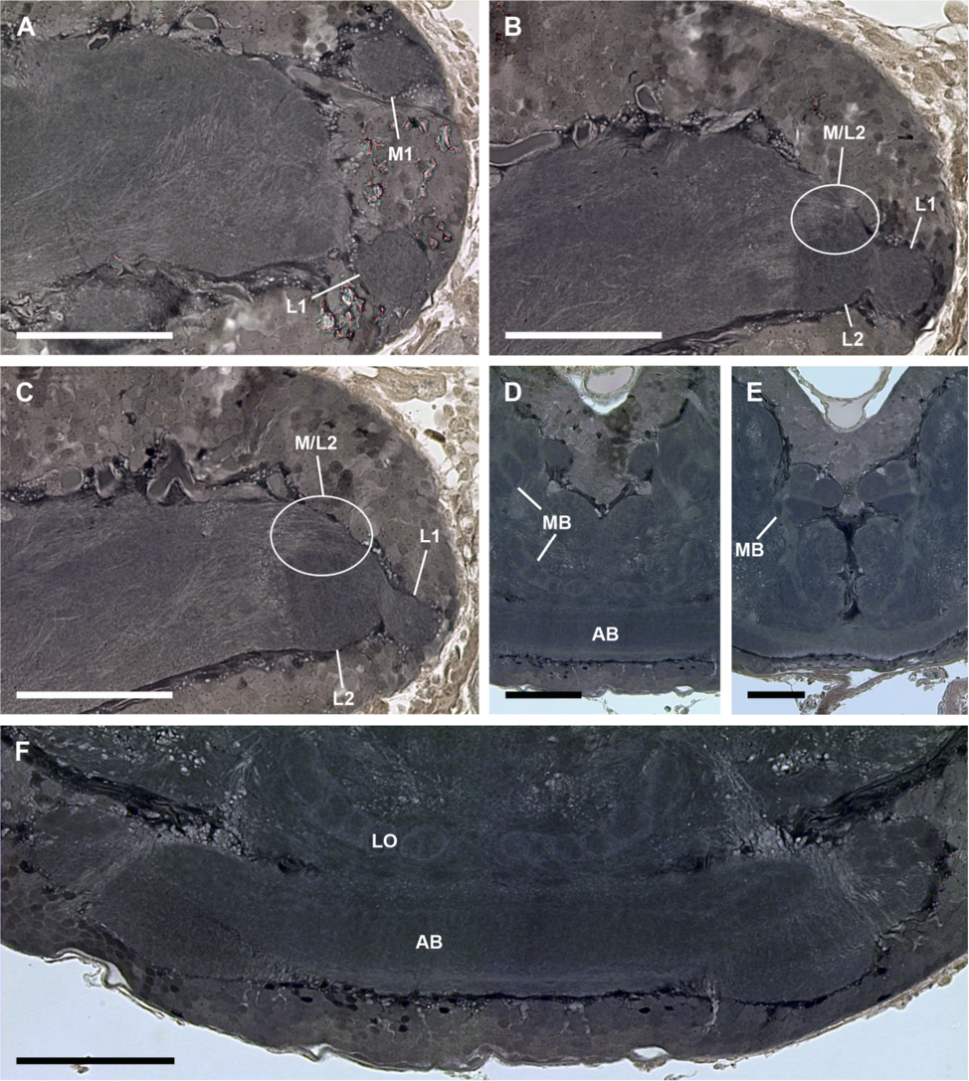
**General anatomy of visual neuropils and protocerebrum (Wigglesworth stains).** Note dark stain of sensory neuropils after application of Wigglesworth’s technique. Bars 100 μm. **A**, first visual neuropils of median and lateral eyes, sagittal section. **B**, **C**, first and second visual neuropils of lateral eyes. Encircled: region where also R-cell axons of median eyes terminate, sagittal sections. **D**, **E**, mushroom bodies located parallel to midline of protocerebrum, frontal section. **F**, arcuate body in dorso-posterior position, frontal section. AB, arcuate body; L1, first lateral eye visual neuropil; L2, second lateral eye visual neuropil; M1, first median eye visual neuropil; M2, second median eye visual neuropil; M/L2, region of L2 or M2 with Cobalt-filled R-cell axons of both median and lateral eyes; MB, mushroom bodies.

The visual neuropils are unequivocally identified with Cobalt fills and DiI / DiO labelling (Figures 1, 2, 3 and 4), and can also be recognised with osmium-ethyl-gallate staining (Figure 5), as dark-stained areas, as is typical for dense neuropils such as sensory neuropils. The third target region, i.e. that of the median eyes in the vicinity of the arcuate body, is also identified with both, Cobalt fills and DiI / DiO labelling (Figures 1, 4).

Furthermore, the arcuate body occupies a superficial, dorso-posterior position in the brain; its shape is slightly bent anteriorly (Figure 5F). The mushroom bodies are located parallel to the midline on each side of the protocerebrum (Figure 5D, E). Both neuropils can be recognised with osmium-ethyl-gallate staining.

### Cobalt fills and DiI labelling via median eyes

Both methods of staining via the median eyes reveal two distinct retinula axon target regions in each hemisphere of the protocerebrum, a first and a second visual neuropil (Figures 1, 4A–C). Furthermore, fibres attributed to visual neurons connect the median eyes with the arcuate body (Figures 1, 4A–C).

Cobalt fills: Immediately after entering the brain the retinula axons build synaptic varicosities all over their extension within the neuropil (Figure 1A). After the first neuropil the retinula axons project ventro-posteriorly in a tract through the cell body rind deeper in the protocerebrum (Figure 1A, B). In this tract no synaptic varicosities appear. After passing through the cell body rind the axons diverge in two directions (Figure 1B, D). The larger parts of the axons first make a U-turn, then project anteriorly towards the visual neuropils of the lateral eyes (see below), while a few axons run further posteriorly to the vicinity of the arcuate body – which lies dorso-posteriorly in the protocerebrum – without entering the arcuate body directly (Figure 1A, B, D, E, G). These posterior-running fibres connecting the median eyes with the arcuate body were observed in a few specimens only. Immediately after the bifurcation, about half a dozen fibres with few synaptic varicosities are visible, but only one or two fibres are Cobalt-filled as far as the vicinity of the arcuate body. This might have resulted from experimental diffusion times (1–4 h) too short for such a long distance (approx. 300 μm). The anteriorly running fibres end in the second visual neuropil (Figure 1B–F). This neuropil lies underneath the cell body rind and is split in two subunits, an anterior and a posterior one, divided by an annulus (Figure 1E, F). Synaptic varicosities occur in both subunits. The anterior subunit lies in the dorsal part of the second visual neuropil of the lateral eyes (see below).

DiI labelling: The same target regions identified with Cobalt fills could be labelled with DiI (Figure 4A–C). After the first neuropil the retinula axons project ventro-posteriorly and diverge in two directions. The larger parts of the axons project to the second neuropil, while few fibres attributed to visual neurons run further posteriorly to the arcuate body. The morphology of the second visual neuropil is very similar to that visible in the Cobalt fills (Figure 4B). Again the neuropil is composed of two subunits divided by an annulus. However, the two subunits extend more ventrally; in the Cobalt fills, synaptic varicosities of the anterior subunit can be found only in the dorsal part of the second visual neuropil of the lateral eyes, while with DiI labelling synaptic varicosities can be found throughout this neuropil. This may be a result of the long diffusion time and hence of transcellular labelling. The fibres running posteriorly towards the arcuate body can be identified with DiI labelling as well. Synaptic varicosities after the bifurcation are better recognisable than in the Cobalt fills. Furthermore, the axons are labelled all the way through the arcuate body, the pale labelling resulting from the fact that only few axons are present in this area (Figure 4A).

### Cobalt fills via lateral eyes

Cobalt fills via the lateral eyes also reveal two distinct retinula axon target regions in each hemisphere of the protocerebrum, a first and a second visual neuropil (Figure 2).

After entering the first visual neuropil the retinula axons build synaptic varicosities all over their extensions (Figure 2A–C). A chiasma between the first and second visual neuropils is not positively identified in any of the chosen section planes (sagittal, frontal or transversal), but fibres that seem to cross between first and second visual neuropil are observed (Figure 2D, E).

In the second visual neuropil the retinula axon terminals are branched and have synaptic varicosities (Figure 2D, E). In the dorsal region of this neuropil terminals of the retinula axons of the median eyes are observed in preparations in which retinula axons of both median and lateral eyes are Cobalt-filled (Figure 3B, D) (see below).

### Cobalt fills and DiI / DiO labelling simultaneously via median and lateral eyes

As above the median eyes are directly linked to a first and a second neuropil, and connected to the arcuate body (Figures 1, 3, 4), the lateral eyes are linked to a first and a second neuropil (Figures 2, 3, 4E ,F).

Cobalt fills: The second visual neuropils of median and lateral eyes overlap each other. This means that besides the regions with only Cobalt-filled R-cell axons of median or lateral eyes, there is a region with Cobalt-filled R-cell axons from both median and lateral eyes (Figure 3B, D).

DiI / DiO labelling: The second visual neuropils of median and lateral eyes overlap each other, and the same region with R-cell axons from both median and lateral eyes can be identified with DiI labelling via lateral eyes and with DiO labelling via median eyes (Figure 4E). DiI- and DiO-labelled cell bodies in the cell body rind near the neuropils indicate that transcellular labelling occurred (Figure 4D–E). Hence, in contrast to the Cobalt fills, where no transcellular staining occurred, DiO from the median eyes is identifiable even in the first lateral eye neuropil, and DiI from the lateral eyes even in the posterior subunit of the second median eye neuropil.

**Figure 6:**
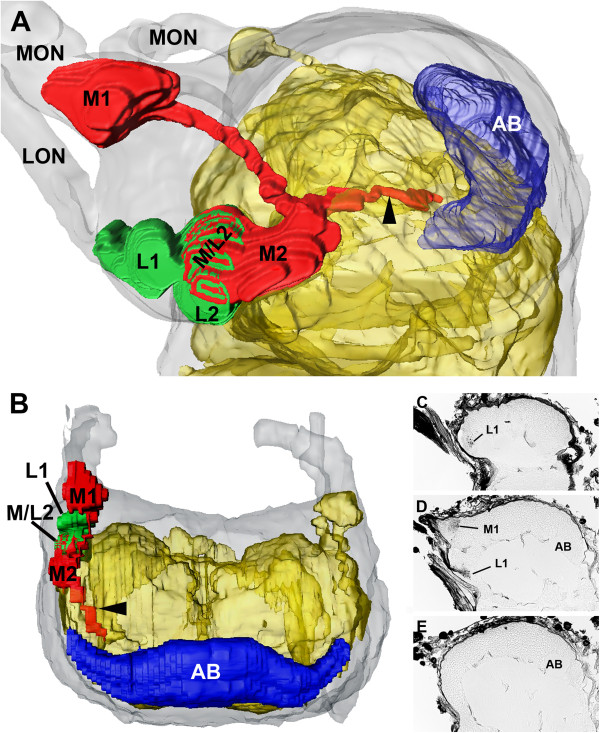
**3D serial reconstruction of visual system of left hemisphere in *E. italicus *on the basis of Cobalt fills. A**, **B,** 3D reconstruction showing arrangement of neuropils. **A**, lateral view; **B**, frontal view. Grey, protocerebrum; yellow, neuropil; red, median eye neuropils; green, lateral eye neuropils; blue, arcuate body. **C**–**E**, three selected sections (Cobalt fills) showing original data for reconstruction. **C**, parasagittal section showing beginning of first lateral eye visual neuropil. **D**, parasagittal section showing first median and lateral eye visual neuropils, and beginning of arcuate body. **E**, mid-sagittal section without visual neuropils but with arcuate body. AB, arcuate body; LON, lateral eye optic nerve; L1, first lateral eye visual neuropil; L2, second lateral eye visual neuropil; MON, median eye optic nerve; M1, first median eye visual neuropil; M2, second median eye visual neuropil; M/L2, region of L2 or M2 with Cobalt-filled R-cell axons of both median and lateral eyes.

Figure 6 shows the summary of the retinula axons and visual neuropils of the median and lateral eyes in *E. italicus.*

## Discussion

The present study constitutes another case that a comparison of more recent findings with those of the early 20th century neuroanatomists, Nils Holmgren [7] and Bertil Hanström [8] is worthwhile. The latter authors correctly identified the visual neuropils of scorpions, but misinterpreted the tracts between them. Holmgren described the same neuropils as Hanström, but did not differentiate between median and lateral eye neuropils. Holmgren’s first and fourth visual neuropils actually are median eye neuropils, his second and third neuropils are lateral eye neuropils. Hanström made this differentiation, but contrary to what he suggested, the median eyes are associated with two subsequent visual neuropils (not only with one), and the lateral eyes are associated with two subsequent visual neuropils (not with three). Hanström described a tract connecting the median eye neuropil with a third lateral eye visual neuropil. In our results that tract is the one connecting the first with the second median eye neuropil; Hanström may have been misled by the position of the second median eye neuropil to misinterpret the latter as a third lateral eye neuropil. In addition, a connection between the visual system and the arcuate body was observed by both authors, which can be confirmed here.

Our results show that the median eye retinula cells are linked to a first and a second visual neuropil, while some fibres additionally connect the median eyes with the arcuate body. The lateral eye retinula cells are linked to a first and a second visual neuropil as well. Furthermore, our stainings show that there is a region in which the second median and second lateral eye neuropils overlap each other. One can distinguish three regions (from posterior to anterior): (1) a region with R-cell axon terminals of median eyes only, (2) a region with R-cell axon terminals of both, median and lateral eyes, and (3) a region with R-cell axon terminals of lateral eyes only. This division is particularly evident in the Cobalt fills. In the DiI and DiO labelling transcellular staining occurred. The latter is recognisable by the fact that cell bodies of interneurons are labelled. Hence, the division of these three regions is visible but not as distinct as in the Cobalt fills, where no transcellular staining occurred. There are three alternative ways to describe and name these regions. One may consider this region as one neuropil, as two neuropils overlapping each other, or as three neuropils (one median, one median/lateral, and one lateral eye visual neuropil). We prefer the second alternative and consider this region as two neuropils, one second median eye neuropil and one second lateral eye neuropil, which partly overlap each other. This means that there is a region with R-cell axons of both median and lateral eyes, but in both second visual neuropils there are also regions with only retinula axon terminals of median or lateral eyes. Moreover, the retinula axon terminals of the lateral eyes are described here for the first time: R-cell axon terminals are found in a first and a second lateral eye neuropil. The crossing fibres we observed probably do not represent a “classical” chiasm as found in Tetraconata [12,33]. A detailed analysis is needed to find out if it might correspond to the chiasm in *Limulus*, which is suggested to be convergent to that in Tetraconata [15,34].

The only other more recent surveys considering the morphology of the visual systems in scorpions were made by Fleissner [9] and Heinrichs and Fleissner [35]. These studies discussed mainly the electrophysiology of the scorpion visual system, and gave only schematic drawings of the approximate locations of the visual neuropils without identifying the latter. However, Fleissner [9] and Heinrichs and Fleissner [35] did report that the different cell types of the median eye retina have different target regions: the photoreceptor cells terminate in a first neuropil (lamina), the arhabdomeric cells in a second neuropil (medulla) [9], while the efferent neurosecretory fibres have their origin/cell body in the tritocerebrum and terminate, while passing through the arcuate body, in the retina of the median eyes [35]. The target region of the photoreceptor cells is located where we found the first median eye neuropil, and the target region of the arhabdomeric cells is where we found the second median eye neuropil; the pathways of the neurosecretory fibres are equal to the fibres we found that connect the median eyes with the arcuate body. Such differentiation of target regions of the different cell types could not be achieved with the methodology chosen for the present study, but will be considered in the discussion below.

Thus our study, while taking the results of Fleissner and Heinrichs into account, leads to a new interpretation of the visual system as well as of the general architecture of the scorpion protocerebrum. The median eyes are associated with two serial neuropils, a first and a second visual neuropil, while some fibres connect the median eyes with the arcuate body. The second visual neuropil is subdivided by an annulus; the posterior subunit contains only retinula axon terminals of the median eyes, while the anterior subunit contains retinula axon terminals of both median and lateral eyes. Furthermore, Fleissner [9] showed that the first neuropil is the target region of the photoreceptor cells, and the second visual neuropil that of the arhabdomeric cells. The morphology of the fibres projecting to the arcuate body is very similar to that of the efferent neurosecretory fibres described by Heinrichs and Fleissner [35]. The authors identified efferent neurosecretory fibres with cell bodies in the tritocerebrum projecting through the arcuate body to the retina of the median eyes. Hence, the fibres projecting to the arcuate body, observed here in Cobalt and DiI stains, are rather retrograde-filled axons projecting from the tritocerebrum through the arcuate body to the retina of the median eyes. Due to the fact that these cells have their cell bodies in the tritocerebrum, they rather “belong” to the brain and are not retinula cells.

The lateral eyes are associated with two serial neuropils, a first and a second visual neuropil. Retinula axon terminals occur in both neuropils, while in the dorsal part of the second visual neuropil retinula axon terminals of both lateral and median eyes are observed.

The slightly bent arcuate body is shown in a superficial, dorso-posterior position in the brain, as is typical for chelicerates [36]. Additionally the mushroom bodies can be observed, located parallel to the midline of the protocerebrum.

These highly specific features described in the present study allow a comparison with the visual systems in other chelicerates and in ancestral arthropods.

### Median eyes

In *Limulus* one must distinguish between the paired median eyes and the fused median rudimentary eye (see review by Battelle [16], and Table 1). Chamberlain and Barlow [13] demonstrated by means of Cobalt fills that the median optic nerve, which contains fibres from both, the paired median eyes and the fused median rudimentary eye, is linked to the first median eye neuropil (ocellar ganglion), arcuate body, optic tract, and medulla (which is also the second lateral eye neuropil). Additionally Calman et al. [14] and Battelle [16] showed with antibody staining that the photoreceptor cells of the paired median eyes are linked in each brain hemisphere only to the first median eye neuropil (ocellar ganglion). Moreover, the authors derived the projections of the arhabdomeric cells by subtracting the photoreceptor cell projections from the results of Cobalt fills of the median eye nerve in Chamberlain and Barlow [13]. According to Calman et al. and Battelle the arhabdomeric cells end only in the medulla (second neuropil of the lateral eyes), but if one compares the results of Calman et al. [14] and Battelle [16] with those of Chamberlain and Barlow [13] one can see that Calman et al. and Battelle ignored that numerous collaterals can be found not only in the medulla (second lateral eye neuropil) but also in the optic tract before entering the medulla. Hence, one can see the target region of the arhabdomeric cells as a neuropil of its own that partly overlaps with the medulla (second lateral eye neuropil). This situation is very similar to the situation found here for the median eyes of scorpions: the second visual neuropil as a target of the arhabdomeric cells partly overlaps with the second lateral eye neuropil as well.

**Table 1:** **Distribution of eyes in Onychophora and Chelicerata**[1,5,16,22,37]

	**Median eyes**	**Lateral eyes**
**Onychophora**	One pair of eyes (median/lateral affinity unknown)	
**Pycnogonida**	Four	Absent
**Xiphosura**	One pair, plus one fused median rudimentary eye	One pair of lateral compound eyes, plus one pair of lateral rudimentary eyes
**Scorpiones**	One pair	Three to five pairs, plus one pair of nymphal eyes
**Araneae**	One pair (= principal eyes or anterior median eyes)	Three pairs (= secondary eyes)

Calman et al. [14] and Battelle [16] also demonstrated with biocytin injection and myosin III immunoreactivity that the fused median rudimentary eye of *Limulus* is linked in each brain hemisphere to the first median eye neuropil (ocellar ganglion) and simultaneously to a region near the arcuate body. This situation is similar to the median eyes of pycnogonids: their eyes are associated with a first visual neuropil and a second visual neuropil in close vicinity to the arcuate body [21]. However, the retinula axons of the fused median rudimentary eye in *Limulus* have some branches in both, the first median eye neuropil and the region near the arcuate body. In pycnogonids the retinula axons have branches only in the first or second visual neuropil, not in both neuropils simultaneously.

In Araneae there is only one target region of the retinula axon terminals of the median eyes (principal eyes or anterior median eyes): the first anterior median eye neuropil, located dorso-laterally in each brain hemisphere [18,19]. Subsequent second-order neurons terminate in a second visual neuropil (medulla); furthermore, a tract that extends into the arcuate body is suggested. Comparing the projections of the median eyes in scorpions with those of the anterior median eyes in Araneae, one finds similarities and differences. The photoreceptor cells project only to a bilaterally paired first visual neuropil. Furthermore, only photoreceptor cells and no arhabdomeric cells are described from the retinae of spiders. Hence, a connection from these cells to a second visual neuropil is missing.

Finally, in Onychophora (*Euperipatoides rowelli*) – one of the suggested sister taxa of Euarthropoda [38,39] whose brain organization is discussed as being similar to that in chelicerates [23,40] – the presence of photoreceptor terminals in a first visual neuropil, which lies directly beneath the eye, is suggested [22]. From this first neuropil, an optic tract projects further and then bifurcates [23]. Its ventral branch extends to a second visual neuropil near the mushroom body calyces, while the dorsal branch gives rise to another second visual neuropil, which flanks the arcuate body laterally. The exact projection of the retinula cells is not identified unequivocally.

Thus, comparing the median eye visual system in scorpions to those in other chelicerates and in onychophorans, there are great similarities to the “normal” median eyes of xiphosurans, and some to the median rudimentary eyes of xiphosurans and median eyes of onychophorans, pycnogonids and spiders. As demonstrated in Lehmann et al. [21], the eyes of pycnogonids and the fused median rudimentary eye of *Limulus*, possibly also the eyes of onychophorans, show striking similarities in their innervation patterns.

The same is true for the median eyes of scorpions and *Limulus*. Both have two distinct, bilaterally paired target regions of the retinula cells: a first neuropil as target for the photoreceptor cells, and a second neuropil, which overlaps with the second neuropil of the lateral eyes, as target for arhabdomeric cells [13,14].

### Lateral eyes

Of special interest here are the eyes of *Limulus*, where one must distinguish again between the lateral rudimentary eyes and the lateral compound eyes (see review by Battelle [16], and Table 1). The rudimentary eyes are associated with the same neuropils as the lateral compound eyes, a first (lamina) and a second (medulla) visual neuropil; the second neuropil is also a target region of the arhabdomeric cells of the median eyes (see above) [14]. While the photoreceptor cells of the rudimentary eyes are linked to both, lamina and medulla, the photoreceptor cells of the lateral compound eyes are linked to the lamina only. Moreover, the retinae of the lateral compound eyes contain eccentric cells, which project to the lamina, medulla, optic tract, and to the first neuropil of the median eyes (ocellar ganglion).

Hence, the projections of the lateral eyes of scorpions have some characters in common with the lateral rudimentary eyes of *Limulus*. Like the lateral eyes of scorpions, the rudimentary eyes have projections to a first and a second visual neuropil. In turn, the photoreceptor cells in the lateral compound eye of *Limulus* are linked to the lamina only, while the eccentric cells are linked to the lamina and medulla of the lateral eye, optic tract, and to the first neuropil of the median eyes (ocellar ganglion). Such a connection from the lateral eye to median eye neuropils cannot be observed in the scorpion visual system. The similarity in function and structure between the eccentric cells and the arhabdomeric cells of scorpions was discussed by Schliwa and Fleissner [3,41]. More research has to be done to distinguish between the exact innervation patterns of the photoreceptor cells and the arhabdomeric cells in the lateral eyes of scorpions.

## Conclusions

**Figure 7:**
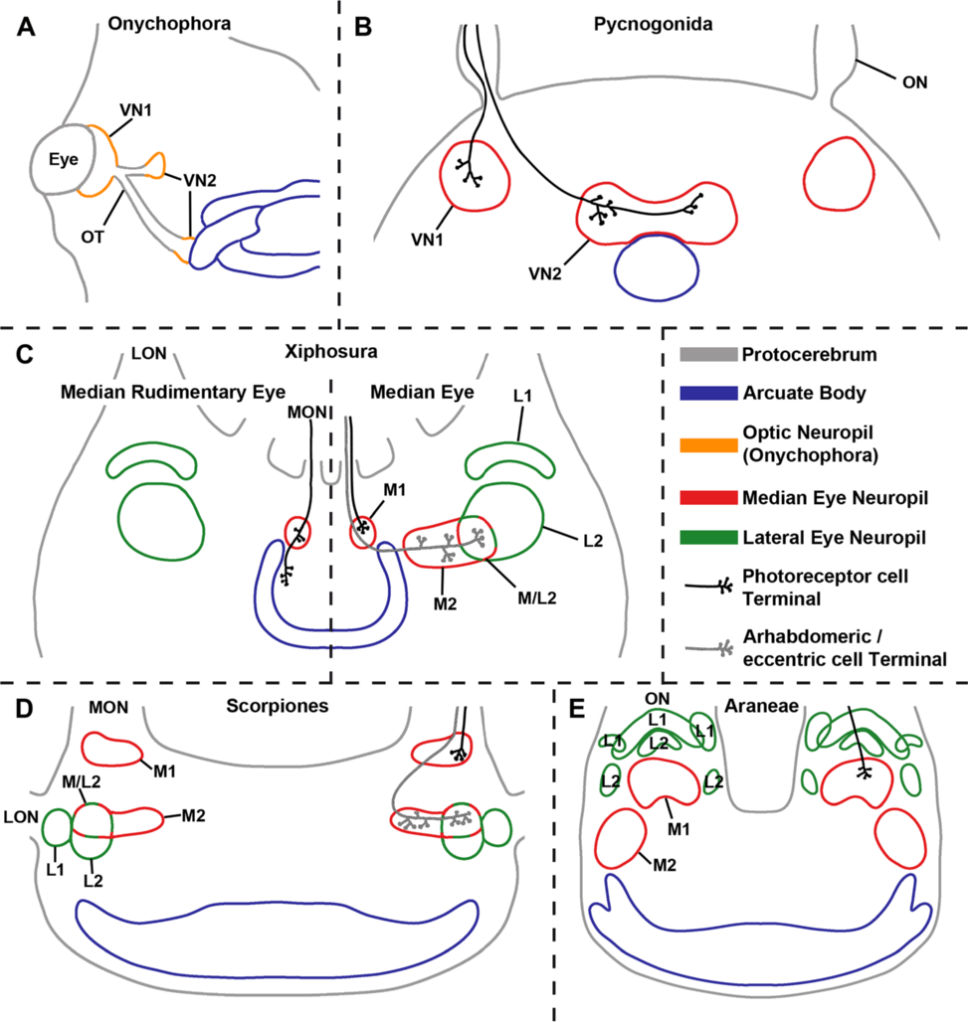
**Comparison of median eye visual systems in (A) Onychophora (*Euperipatoides rowelli*), (B) Pycnogonida (*Achelia *spp.*, Endeis spinosa*), (C) Xiphosura (*Limulus polyphemus*), (D) Scorpiones (*Euscorpius *spp.*, Androctonus australis*), and (E) Araneae (*Cupiennius salei*). A**, visual pathways from eyes with optic neuropils indicated. After Strausfeld et al. [23]. **B**, retinula cells terminate in first and second visual neuropils. Second visual neuropil in close vicinity to an unpaired midline neuropil, possibly an arcuate body. After Lehmann et al. [21]. **C**, left: terminals of median rudimentary eye have some branches in first median eye neuropil, then continue and terminate near arcuate body; right: median eye photoreceptor cells terminate in first median eye neuropil, arhabdomeric cells in second median eye neuropil, which partly overlaps with second lateral eye neuropil. After Calman et al. [14] and Chamberlain and Barlow [13], second median eye neuropil added (see text). **D**, photoreceptor cells terminate in first median eye neuropil, arhabdomeric cells in second median eye neuropil, which partly overlaps with second lateral eye neuropil. Connection between median eyes to region near arcuate body omitted. According to Heinrichs and Fleissner [35] these fibres belong to neurosecretory cells with origin in the tritocerebrum, hence are cells of the brain rather than retinula cells. **E**, retinula cells terminate in first median eye neuropil. After Strausfeld et al. [18] and Strausfeld and Barth [19]. LON, lateral eye optic nerve; L1, first lateral eye visual neuropil; L2, second lateral eye visual neuropil; MON, median eye optic nerve; M1, first median eye visual neuropil; M2, second median eye visual neuropil; M/L2, region were M1 and L1 overlap; ON, optic nerve; OT, optic tract; VN, visual neuropil.

The large number of characters discussed in this article shows that the central projections of especially the median eyes in Chelicerata provide structures that are extremely useful for discussing aspects of chelicerate ground patterns and phylogenetic relationships. The sets of characters studied here for Scorpiones and those in *Limulus*, Pycnogonida, Onychophora, and Araneae are summarised in Figure 7.

As shown above, the similar innervation patterns of the median and lateral eyes indicate a close relationship concerning the visual system between scorpions and *Limulus*. Other characters supporting this idea are the position and cellular architecture of the accessory lateral eye of scorpions, which corresponds well with that of the lateral rudimentary eye of *Limulus*[5]. Also the functional and structural similarity of the arhabdomeric cells of scorpions with the eccentric cells of *Limulus* lateral eyes must be mentioned [3,41]. Dunlop and Webster [31] discuss further similarities between scorpions and *Limulus*. Besides similar sperm morphology and growth zones, the shared character of star-shaped rhabdoms is mentioned (see also Weygoldt and Paulus [24]). However, the argument of rhabdom morphology must be handled with care: indeed, scorpions and *Limulus* both have star-shaped rhabdoms, but this is only true for the lateral compound eyes of *Limulus* and the median but not the lateral eyes of scorpions. The latter have a net-like rhabdom [3]. Nevertheless, characters of the visual system support the hypothesis of Weygoldt and Paulus [24] that scorpions occupy the basalmost position within Arachnida, or even the idea of palaeontologists that Scorpiones are closely related to Eurypterida [31,32] and hence also to Xiphosura. This, in turn, would question the monophyly of Arachnida, and would mean that scorpions and one or more other arachnid lineages are likely to have come onto land independently [31]. More research concerning the visual systems in Arachnida has to be done, since only few taxa have been investigated, and there are no data on various taxa like Opiliones, Pseudoscorpiones, and Solifugae, which are suggested as sister taxa to Scorpiones by various authors [25-28].

Regarding the basal position of *Limulus* and especially Pycnogonida, it is reasonable to assume that the central projections of the median rudimentary eye in *Limulus* and the four median eyes in Pycnogonida represent the ground pattern for Chelicerata. This ground pattern is characterised by (1) four median eyes, (2) a separated, bilaterally paired nerve that connects the eyes with the brain, (3) a separated, bilaterally paired first visual neuropil with central projections of photoreceptor cells, (4) a second visual neuropil also with central projections of photoreceptor cells, and (5) the second visual neuropil being located in close vicinity to the arcuate body. Derived situations are found in the “normal” median eyes of *Limulus* and in the median eyes of scorpions: in both of these, the photoreceptor cells only project to a separated, bilaterally paired first visual neuropil, while the second type of retinula cells, the arhabdomeric cells, project to a second visual neuropil, which partly overlaps with the second visual neuropil of the lateral eyes. Additionally a third cell type is found in the retina of the median eyes, the efferent neurosecretory fibres, which have their origin/cell body in the brain and terminate in the retina. Another derived situation is found in the median eyes (principal eyes or anterior median eyes) of Araneae, whose photoreceptor cells (as the only cells in the retina projecting to the protocerebrum) simply project to a separated, bilaterally paired first visual neuropil.

## Materials and methods

The use of *Euscorpius* spp. in the laboratory doesn't raise any ethical issues and therefore Regional or Local Research Ethics Committee approvals are not required.

### Specimen collection

Specimens of *Euscorpius italicus* (Herbst, 1800) (Scorpiones: Euscorpiidae) were collected during field trips to Rovinj (Croatia) in August 2011 and April 2012. Specimens of *Euscorpius hadzii* Di Caporiacco, 1950 were provided by b.t.b.e. Insektenzucht GmbH (Schnürpflingen, Germany).

### Cobalt fills

(*Euscorpius italicus*, modified after Altman and Tyrer [42]): CoCl_2_ crystals were inserted in median, lateral, or median and lateral eyes with a fine tungsten needle (n = 30). After diffusion times between 1 and 4 hours, Cobalt was precipitated with a solution of five drops of (NH_4_)_2_S in 10 ml H_2_O_dest_. After fixation of the cephalothorax in AAF (85 ml 100% ethanol, 10 ml 37% formaldehyde, 5 ml glacial acetic acid), the brain was dissected and silver intensified: 60 min at 50°C in dark in solution A (10 ml H_2_O_dest_, 3 ml 100% ethanol, 0.5 g gum arabic, and 0.02 g hydroquinone; pH value adjusted to between 2.6 and 3.1 using citric acid), and 15–30 min at 50°C in the dark in solution B (10 ml H_2_O_dest_, 3 ml 100% ethanol, 0.5 g gum arabic, 0.02 g hydroquinone, 0.01 g AgNO_3_; pH value adjusted to between 2.6 and 3.1 using citric acid). Silver intensification was stopped in an acetic acid solution (50 ml 30% ethanol, 5 g glucose, pH value adjusted to between 2.6 and 3.1 using acetic acid). After dehydration in a graded acetone series, the brain was embedded in Glycidether 100, and sectioned with a rotary microtome and stainless steel blade in the sagittal, frontal, and transversal planes (14–16 μm).

### DiI / DiO labelling

(*Euscorpius hadzii*, after Wohlfrom and Melzer [43]): The cephalothorax was dissected and fixed overnight at 4°C in 4% formaldehyde in 0.1 M PBS. Afterwards specimens were rinsed overnight in 0.1 M PBS, 0.1% NaN_3_. Finally, small DiI or DiO crystals (Molecular Probes) were inserted in median or median and lateral eyes with a fine tungsten needle. Diffusion was carried out in darkness on small glass slides enclosed in wet chambers for 17–22 days. To prevent the growth of microorganisms, NaN_3_ in PBS was used for moistening. From time to time the specimens were controlled under the microscope. Specimens were studied with a fluorescence microscope and CLSM.

### Osmium ethyl gallate procedure

(*Euscorpius italicus*, modified after Wigglesworth [44], Leise and Mulloney [45], and Mizunami et al. [46]): Brains were dissected and fixed in 4% glutardialdehyde in 0.1 M cacodylate buffer at 4°C (n = 7). After postfixation in 2% OsO_4_ in 0.1 M cacodylate buffer (3 h at 4°C) animals were stained for 17 hours at 4°C in a saturated ethyl gallate solution, dehydrated in a graded acetone series, embedded in Glycidether 100, and sectioned with a rotary microtome and stainless steel blade in the sagittal, frontal, and transversal planes (5–8 μm).

### 3D-reconstruction

Brain (prepared as for Cobalt fills) was cut into a complete sagittal series (16 μm). Slices were mounted on glass slides, covered with cover slips, and photographed under a conventional light microscope. Images were contrast-enhanced in Adobe Photoshop, then aligned, segmented and rendered in Amira.

## Competing interests

The authors declare that they have no competing interests.

## Authors’ contributions

TL conceived the study, carried out the morphological analysis, and drafted the manuscript. RRM conceived and supervised the study and helped writing the paper. All authors read and approved the final manuscript.
